# Delivering treatment to morally injured UK military personnel and Veterans: The clinician experience

**DOI:** 10.1080/08995605.2021.1897495

**Published:** 2021-03-29

**Authors:** Victoria Williamson, Dominic Murphy, Sharon A. M. Stevelink, Shannon Allen, Edgar Jones, Neil Greenberg

**Affiliations:** a King’s Centre for Military Health Research, Institute of Psychology, Psychiatry and Neuroscience, King’s College London, London, UK; b Research Department, Combat Stress, Leatherhead, UK; c Department of Psychological Medicine, Institute of Psychology, Psychiatry and Neuroscience, King’s College London, London, UK

**Keywords:** Moral injury, mental health, psychological treatment, clinician, qualitative methods, military

## Abstract

This study explored the experiences of clinicians in providing treatment in cases of military-related moral injury (MI). Qualitative interviews were carried out with 15 clinicians. Clinicians found patients experienced particular maladaptive appraisals following MI, which were considered different from the responses experienced after threat-based trauma. To address MI-related distress, clinicians utilized a range of treatment approaches. Several difficulties in providing care to patients following MI were described, including the impact of providing treatment on the clinicians own mental health. This study provides detailed insight into the approaches currently used to identify and treat UK Veterans with MI-related psychological problems. These findings highlight the need to evaluate the effectiveness of the treatments currently provided for MI-related psychological problems and suggest developing best practice guidance may improve clinician confidence in delivering care to those adversely impacted by MI.

**What is the public significance of this article?**—This research provides insight into the experiences of clinicians providing care to patients affected by moral injury-related psychological problems. We detail the treatment approaches currently utilized and difficulties faced when working with morally injured patients. Future research is needed to evaluate the treatments currently provided following moral injury.

## Introduction

Exposure to potentially morally injurious events (PMIEs), defined as acts of omission or commission that transgress deeply held moral beliefs, can cause significant psychological distress (Litz et al., 2009). This distress has been termed “moral injury” and is associated with some forms of mental illness, including posttraumatic stress disorder (PTSD), depression and suicidal ideation (Williamson, Stevelink, & Greenberg, 2018). Research suggests the psychological distress experienced following PMIEs may represent a distinct symptom profile involving moral emotions of guilt and shame as well as depressive-type symptoms, compared to the more intrusive and hyperarousal symptoms defining the PTSD profile (Griffin et al., 2019). Given the nature of PMIEs, individuals with mental health problems (e.g. PTSD, depression) related to morally injurious events may be reluctant to seek formal support due to concerns about the social/legal consequences of disclosure. For example, patients may be uncertain about the limits of clinician-patient confidentiality and be concerned whether their disclosure of a PMIE could lead to an investigation into their actions by authorities (Frankfurt & Frazier, 2016; Williamson et al., 2020). Further complexity is added because for those patients who do seek support, no standardized treatment for moral injury-related mental health problems currently exists (Griffin et al., 2019; Maguen et al., 2010). Moreover, it is also possible that that some standard treatments for PTSD, such as exposure-based treatments (e.g. prolonged exposure), alone may not adequately address all negative sequelae present in those affected by moral injury (Maguen & Burkman, 2013).

How clinicians treat moral injury-related mental health problems in a UK context has received limited research attention to date. A recent pilot study found that clinicians use an amalgamation of several manualised PTSD treatments for UK military Veterans affected by moral injury (Williamson, Greenberg, & Murphy, 2019). Although, as this study only included the views of four clinicians, all of whom worked at a center specializing in Veteran PTSD treatment, this may limit the generalizability of these findings. It is also possible that clinicians may experience a greater degree of vicarious distress when treating patients with moral injury-related mental health problems (Griffin et al., 2019). Exposure to PMIEs often generates strong negative emotions, leading to a loss of trust in the world and disillusionment with humanity generally (Frankfurt & Frazier, 2016). Some clinicians may not feel adequately prepared to manage the range of issues with which patients with moral injury present (McCormick et al., 2017). A better understanding of presentations, clinical approaches and potential barriers to care may ensure that appropriate treatment and support are available to those with mental health difficulties following exposure to PMIEs. We carried out in-depth qualitative interviews with clinicians who provide psychological treatment to UK military personnel and Veterans. This study explored the experiences and challenges faced by clinicians in providing treatment in cases of moral injury-related mental health problems to inform potential improvements to the provision of psychological care.

## Methods

### Ethical approval

This study received ethical approval from King’s College London Research Ethics Committee (RESCM-17/18-4002).

### Participants

Between October 2018 and January 2019, 15 clinicians were recruited. Inclusion criteria were having provided psychological treatment to a UK military personnel or Veteran within the last six months whom the clinician considered had experienced moral injury-related mental health problems. Participants were purposively sampled from a range of clinicians who had worked, or were working, with the National Health Service (NHS), Ministry of Defense (MoD), and voluntary sector organizations. We employed a snowball sampling methodology. To recruit participants, e-mails were sent to therapists responsible for providing trauma therapy across collaborating organizations (e.g. Combat Stress, Walking with the Wounded) as well as circulating study advertisements via mailing lists, social media and in Veteran-affiliated newsletters. Participating clinicians were also asked to share the study with potentially eligible colleagues. Of the 21 clinicians approached, 15 (71%) consented. Five clinicians were uncontactable, and one was not eligible to participate having not practiced as a clinician for several years.

### Qualitative interview schedule

Interviews were conducted by a researcher who had training and experience in qualitative methods (VW). Thirteen interviews were carried out by telephone, two were conducted in person. All participants gave written (if interview conducted in person) or audio-recorded verbal (if interview conducted via telephone) informed consent for their participation.

The interview schedule was developed based on the research questions and the existing literature relating to moral injury (Frankfurt & Frazier, 2016; Williamson et al., 2018). The interview schedule was piloted with four clinicians prior to data collection, pilot data was not included in the present study. Interview questions explored clinician perceptions of military-related moral injury experienced by UK personnel/Veterans, the impact of exposure to moral injury on wellbeing, challenging symptom presentations in patients experiencing moral injury, potential risk and protective factors for military moral injury, and views regarding necessary changes to clinical practice or policy to address moral injury and improve outcomes (Supplementary Figure 1). With consent, interviews were audio-recorded and transcribed verbatim with personally identifying information removed. Prior to the interview, basic demographic information was collected from each participant.

### Analysis

Data were analyzed using thematic analysis. NVivo V.10 software was used to facilitate analysis. The steps for inductive thematic analysis as described by Braun and Clarke (2006) were followed: researchers familiarized themselves with the transcripts, produced codes, searched for and developed themes, revised and refined themes, and determined connections between the themes which, where applicable, were grouped together under superordinate themes. Data collection and analysis took place simultaneously to allow emerging topics of interest to be investigated further in later interviews and to determine whether thematic saturation had been reached. To ensure reliability, all transcripts, codes, sub-themes and superordinate themes were independently reviewed by authors VW and SA, with any disagreements resolved by a thorough reexamination of the data and discussion. Peer debriefing was regularly carried out, with feedback regarding data interpretation sought from coauthors DM, SAMS, NG & EJ.

## Results

### Descriptive characteristics

The mean age of clinicians was 47.1 years (9.4 SD) and 10 (66.7%) were male. Three (20.0%) participants were psychiatrists, five (33.3%) were psychologists, and seven (46.7%) were mental health nurses. Clinicians were all practicing in a variety of inpatient, outpatient or primary care clinics throughout the UK and had worked in clinical practice with service members or Veterans for an average of 16.5 years (1–32 range). Eight participants had also previously served in the Armed Forces. Of the seven clinicians without prior military service, three (42.9%) had received specific training (e.g. seminar, workshop, etc.) in military ethos, military organization and roles, or other aspects of military culture.

### Qualitative findings

As shown in Table 1, four themes and nine sub-themes emerged from the data, reflecting clinicians’ experiences of providing treatment to (ex-)serving UK military patients following moral injury. Anonymized participant comments are provided to illustrate our findings.

**Table 1.** Themes and sub-themes following thematic analysis

Themes and subthemes
Presentation of moral injury
Experiencing a compromised moral code
Perceived impact of moral injury on personnel/Veteran mental health
Treatment of moral injury-related distress
Identifying moral injury
Treatment approaches
Challenges in providing treatment for moral injury-related psychological problems
Potential improvements to the future treatment of moral injury-related distress
The impact of providing treatment on clinicians Rewarding to deliver care Vicarious distress and the clinician’s own military service Importance of clinical supervision
Perceptions of potential risk and protective factors for moral injury
Types of morally injurious events
Individual risk factors
Potentially protective measures to safeguard against moral injury

### Presentation of moral injury

#### Experiencing a compromised moral code

Clinicians considered that experiences of moral injury arose after events that compromised someone’s moral code or core beliefs about themselves, others or the world. Clinicians thought that PMIEs evoked distress because the incident jarred with strongly held beliefs about what a serviceperson should (or should not) do. Moral injury-related distress was thought to differ from that evoked by threat-based traumas, as PMIEs had the potential to produce intense feelings of guilt, worthlessness or shame rather than feelings of fear or vulnerability. The concept of “moral injury” was believed by clinicians to be a helpful addition to the mental health vocabulary by offering a further way to consider the distress caused by events that may not be “classically” traumatic but lead to emotional conflict.

#### Perceived impact of moral injury on personnel/Veteran mental health

Clinicians reported that personnel/Veterans affected by PMIEs often experienced symptoms of PTSD, including intrusive symptoms and avoidance. Emotional numbness, excessive rumination, low mood and pervasive negative appraisals of themselves (e.g. I am a dreadful person) and others (e.g. other people are untrustworthy, the world is an awful place) were also frequently reported. These negative appraisals were described as markedly different from those associated with fear-based trauma where maladaptive appraisals were threat related (e.g. I am vulnerable, the world is dangerous). Feelings of guilt, shame and worthlessness following PMIEs reportedly contributed toward poor self-care, risk taking as well as self-harming behaviors. Notably, moral injury-related distress was considered by clinicians to be increasingly common in personnel/Veterans presenting for treatment.

I’m seeing an awful lot more patients whose distress is caused not by things such as fear … now it’s more along the lines of guilt and shame … .it’s not the old classic ‘I’m scared, and I haven’t really come to terms with the threat is no longer there’ … . we’re spending an awful lot more time talking to soldiers about beliefs about themselves and judgements, am I good, am I bad, did I do wrong, was I wronged?

### Treatment of moral injury-related distress

#### Identifying moral injury

To identify whether patients had experienced a morally injurious event and been adversely affected by this experience, clinicians reported taking a comprehensive trauma history. Typically, experiences of moral injury were thought to become apparent following the clinician asking questions about the nature of the event, how the person felt about what happened, and what the event said about them as a person or the world more generally. Notably, moral injury-related psychological problems were perceived to be an issue that could easily go unrecognized, leading to inappropriate treatments being recommended and poorer patient outcomes.

[In] moral injury we’re talking about what is the cognition maintaining it and that’s where your trauma assessment, where I would sit down with you … and I start asking you, ‘so what does this mean? What does it say about you?’ That’s when you’ll start to get the real thing behind the trauma … the thing I’m after is … what does that say about you? ‘I could have done more.’

#### Treatment approaches

To effectively address moral injury-related distress, clinicians described the need for a holistic approach which takes into account the patient’s particular needs and difficulties. No consensus between clinicians was observed in terms of the best treatment approach. A number of methods were reportedly used in cases of moral injury-related distress, including eye movement desensitization and reprocessing (EMDR), compassion focused therapy, elements of schema therapy, trauma-focused cognitive behavioral therapy (TF-CBT) and mindfulness. Some clinicians described using an amalgamation of several approaches (e.g. use of compassion focused therapy as well as TF-CBT) depending on a patients’ specific needs and symptomology. The rational for using each approach differed. For example, clinicians using EMDR considered this method to be particularly effective following PMIEs as EMDR facilitates processing of the trauma memory and associated distress, yet the patient does not need to disclose the event to the therapist, safeguarding against re-traumatization. Clinicians described that patients affected by moral injury often required several treatment sessions (range: 12–16+), a number that was thought to be consistent with more complex trauma cases.

We deliver EMDR therapy and we don’t ask them for the narrative … I don’t believe in retraumatising people because the amount of times patients will say, yes, I’ve got to tell my story over and over and over again.

#### Challenges in providing treatment for moral injury-related psychological problems

Clinicians described that many personnel/Veteran patients did not seek formal help for several years following PMIEs, often due to mental health stigma concerns. Many patients only accessed treatment once a crisis point had been reached or at the insistence of family members, a pattern commonly seen in treatment-seeking personnel/Veterans (Murphy, Hunt, Luzon, & Greenberg, 2014). Once treatment had been accessed, difficulties including maladaptive coping strategies, re-traumatization, issues of confidentiality and the need to build a trusting therapeutic relationship were reported. Clinicians also stated that some patients continued to feel ongoing guilt or shame following treatment and were reluctant to change the way they interpreted the event as this was thought to be disrespectful. It was ultimately considered the patient’s decision whether they were ready to reevaluate the event, though discussing this dilemma with patients was reported as helpful.

They don’t actually want to remember something differently … to feel shame about what’s happened is a way of remembering and honouring that memory. … Because [in treatment] you are wanting to update the cognitions and their belief about what happened … sometimes there will be a barrier to that and they won’t want to be cognitively restructured … I’m very blunt with them and that I will basically share that formulation with them.

Another key concern was the potential to re-traumatize patients and some approaches, such as reliving or imaginal exposure, were avoided for this reason. This decision was reportedly informed by clinicians’ experience of working with trauma-exposed patients and concerns were expressed that less experienced therapists may not recognize the need for a different approach in cases of moral injury-related distress. Given the sensitive nature of many of the PMIEs, a strong therapeutic relationship with patients was considered essential for treatment to be effective; though, building a trusting relationship with patients affected by moral injury could be challenging, requiring several treatment sessions. At the same time, where morally injurious events involved disclosure of a crime or an incident outside the rules of engagement, this could present clinicians with an ethical dilemma. For example, a patient may disclose an incident that occurred on deployment but – on further questioning – it may become apparent that the patient has a distorted memory of the event or the clinician may come to doubt the incident occurred at all. However, little consensus was found regarding when breaches of confidentiality may be necessary.

Our take is this, we’re not judge and jury - number one. Number two is we’re not here to tell anybody what is confidential. Number three is you’ve got one vision and one view of what’s supposed to have happened and many times the view they tell you to start with is not what happened at all. So, by the time you end up finishing your therapy it’s not the case, it has not happened … You need to tread very carefully indeed, you mustn’t be judgemental, there must be corroborative evidence, we are not going to report things unless we absolutely need to.

#### Potential improvements to the future treatment of moral injury-related distress

The majority of clinicians described a need for greater awareness of the experience and impact of moral injury in clinical practice. Several clinicians reported that improved awareness could be achieved by clearer guidance on identifying moral injury and how moral injury-related ill-health should be treated. Moreover, additional training on delivering moral injury-specific care was considered as potentially beneficial in improving clinician confidence in managing cases of moral injury. However, training could be prohibitively expensive for some.

There could be more openness in people sharing therapy session content and what they are doing … . the profession could do with being a little more open about what skills are used and how it benefits and when we might do things [like] adaptive disclosure or when we might use compassion focused therapy without it having to cost us hundreds of pounds to attend [a course].

### The impact of providing treatment on clinicians

#### Rewarding to deliver care

Providing care to morally injured personnel/Veterans was described as very rewarding by many clinicians.

Myself I find it very rewarding to be honest … It’s a sort of role where you do get a lot of satisfaction because you can see the changes. When you meet someone for the first time and six months down the line you see massive changes … it’s a huge reward. Massive.

#### Vicarious distress and the clinician’s own military service

At the same time, it could also have a negative impact as hearing about a series of PMIEs was often upsetting and emotionally draining. Furthermore, clinicians who had served in the Armed Forces found that treating patients affected by moral injury could also evoke their own memories of challenges faced during military service.

To have one patient after another where you are listening to guilt and shame wears you down … . This is where the supervision comes in quite handy … but supervision needs to improve in military … We [clinicians] deploy with the [troops] … so a lot of the traumas they were coming to us with were ones we’d been involved with … [and] it’s something that worries us, so I’d be very, very cautious about the trauma I allow my junior [clinician colleagues] to be exposed to.

#### Importance of clinical supervision

To manage this, all clinicians reported receiving regular clinical supervision, together with less formal conversations with colleagues. Nonetheless, several clinicians felt that they would find more supervision or support from their organization helpful in promoting their own wellbeing.

[Providing treatment] can be demanding … [It] can be very draining, and it can be quite challenging sometimes to hear these things and not take them with you after the appointment or after work. So, we use supervision, we use continuing professional development (CPD), we look at our caseloads and the kinds of cases we have on the caseload at any one time … to achieve a balance, if at all possible, between different types of cases.

### Perceptions of potential risk and protective factors for moral injury

#### Types of morally injurious events

Certain events in military service were considered by clinicians to be more commonly associated with experiencing moral injury-related distress, including incidents in which civilians were injured/killed, excessive violence was used, or the rules of engagement were unclear.

#### Individual risk factors

Individual factors such as lower educational attainment, experiences of childhood adversity and feeling unprepared or unaware of the emotional/psychological consequences of making ethically challenging decisions were considered potential risk factors for experiencing distress following PMIEs. Transitioning from the military to civilian life was also thought to play a role in experiencing moral injury-related distress. Clinicians observed that re-joining a civilian environment with societal values that differed considerably to the values present in a military context could cause some Veterans to question their previous deeply held beliefs about themselves as a person, their role in operational tours, and the world in which they live.

I’ve often wondered with moral injury does it develop straight after an event or is it when people transition out? When those social and supportive networks are gone and where you are back in a values-based society which is very different to the values of military life. Is it then that you start to look back and say, ‘oh that doesn’t fit with what I now know?’

#### Potentially protective measures to safeguard against moral injury

To promote wellbeing, some clinicians thought that encouraging individuals to view a PMIE as an opportunity for psychological growth could be adaptive, leading to greater self-awareness, empathy and resilience. Improvements to pre-deployment briefings, including better preparation for the potential moral/ethical conflicts that may be experienced by personnel on deployment, as well as a more thorough operational debriefing following incidents which includes reassurance from leaders that personnel acted correctly and within the rules of engagement (where appropriate), were considered by several clinicians to potentially be a protective measure to lessen distress following PMIEs. Furthermore, chaplaincy was thought to have a role by providing individuals with the opportunity to discuss in confidence the PMIE, the thoughts or feelings this experience may have evoked and how to reconcile or forgive oneself.

I think there needs to be more [of a focus] in the decompression … on the individual’s responsibility and more reassurance given closer to the time that they did as much as they could do … I think [if] those ‘what if’ questions were answered by a position in authority, they would listen to it … I find that our job as therapists working with this is we often end up working on that permission giving and that reassurance that comes from us … and maybe if that came earlier … it might have prevented something.

## Discussion

This study aimed to explore the experiences and challenges faced by clinicians in providing treatment to military personnel/Veterans affected by moral injury. We identified four key themes: the presentation of moral injury, current treatment approaches and potential improvements to care, the impact of providing treatment on clinicians themselves, and potential risk and protective factors for those experiencing military-related moral injury.

Moral injury was considered a helpful construct for conceptualizing the psychological distress caused by events which transgressed a person’s moral code. The impact of PMIEs on mental health was described as being different to that typically caused by a threat-based trauma. These findings are consistent with previous studies of moral injury in both military and nonmilitary samples, with the most common symptoms present in cases of moral injury being intense negative appraisals, intrusive thoughts and self-depreciating emotions (Feinstein, Pavisian, & Storm, 2018; Griffin et al., 2019; Litz et al., 2009). Although it should be noted that some symptoms common after PMIEs (e.g. self-blame and guilt) are also frequently reported by those meeting criteria for PTSD following threat-based trauma. Moreover, moral injury-related mental health difficulties were thought by clinicians to be an increasingly common presentation in UK personnel/Veterans. It is possible that this reflects improved awareness of moral injury amongst UK clinical care teams or it may be that the recent conflicts in Iraq and Afghanistan, characterized by Armed Forces personnel often being unable to distinguish civilians from combatants, presents distinct moral challenges. Moral injury has also been found to be common among US, Dutch and Canadian Veterans seeking treatment for mental health difficulties (Frankfurt & Frazier, 2016; Nazarov, Fikretoglu, Liu, Thompson, & Zamorski, 2018; Schut, De Graaff, & Verweij, 2015). We suggest that there is a need for additional research to determine the prevalence of PMIEs, and their association with mental ill-health, amongst UK personnel and Veterans to better understand its relative importance and to ensure that adequate healthcare provision is accessible.

To identify moral injury, clinicians reported gathering a comprehensive trauma history from patients. Clinicians expressed the view that less comprehensive history-taking would lead to a considerable potential for moral injury to be overlooked. Currently there is no measure of moral injury validated for use in UK personnel/Veterans and the results of this study highlight the pressing need for such a tool for clinical practice. The present study also found that clinicians utilized a variety of standardized treatment approaches to address specific maladaptive responses and appraisals. This suggests that a validated measure to assess patient exposure to PMIEs and moral injury-related mental health difficulties may not only be helpful in improving the detection of moral injury but also determining whether current treatment approaches are effectively addressing symptoms. Whilst several treatment approaches have been found to be effective for PTSD in military populations (Eftekhari et al., 2013; Monson et al., 2006), relatively high non-response rates, patient drop out and poor conceptualization of the fit between proposed therapeutic mechanisms and moral injury has raised concerns whether these approaches are appropriate for all trauma types (Nash & Litz, 2013; Phelps et al., 2018; Schottenbauer, Glass, Arnkoff, Tendick, & Gray, 2008). Emerging evidence indicates that particular types of PMIEs (e.g. perpetration, witnessing, failing to prevent, betrayal) may provoke distinct responses. For example, Litz et al. (2018) recently found that perpetration-based PMIEs were associated with greater levels of guilt, reexperiencing, and self-blame compared to life-threat traumas. Thus, individuals with mental health problems related to a moral injury may have distinct psychological responses and potentially have different treatment needs as a result. Promising early evidence indicates Adaptive Disclosure may be effective in treating moral injury related mental health problems for US patients (Litz, Lebowitz, Gray, & Nash, 2017); whether such an approach is equally effective in a UK context remains unclear. An evaluation of whether using existing interventions leads to long-term improvements in morally injured patient’s maladaptive responses and appraisals, whether certain interventions are more appropriate after particular events or if a validated manual for treating moral injury-related psychological problems is needed, remains outstanding.

Treating cases of moral injury appears to be challenging for clinicians due to concerns about patient re-traumatization, concerns about ineffective coping strategies, and issues relating to confidentiality and rapport. In a UK context, clinical psychologists and social workers are obliged to report patient disclosures of allegations of a serious crime, dangerous or abusive behavior; yet little guidance is currently available to support how clinicians should reach a decision to break patient confidentiality and how to manage the subsequent social/legal consequences breaching confidentiality may have for patients (Williamson et al., 2020). Our findings indicate that clinicians would value more accessible training and resources on the identification and treatment of moral injury-related mental health disorders. Such training may increase the confidence of clinicians working with morally injured samples. On a personal level, providing care for this group of patients can also be distressing for clinicians, particularly if patient’s PMIE was similar to their own experience – which was more likely if the clinician had served in the military themselves. Several factors, including personal trauma history, professional trauma exposure (e.g. deployment to combat zones to provide treatment), inadequate adequate training and lack of accessible peer consultation, have been found to impact the severity of vicarious trauma when working with military referrals (Jordan, 2010). While assessing the effects of vicarious trauma was beyond the scope of this study, these results have implications for mental health organizations in ensuring that clinical care teams providing treatment to patients affected by moral injury have access to adequate peer support, clinical supervision and training resources to safeguard their own wellbeing.

Several factors were thought to increase service personnel and Veterans’ vulnerability for experiencing distress following PMIEs, including event type, childhood adversity, unclear rules of engagement, being unprepared, and transitioning to civilian life. These findings are broadly consistent with the previous literature in military samples; for example, difficulties transitioning from the Armed Forces has been found to be associated with a range of poor mental health outcomes (Diehle & Williamson, 2019; Hatch et al., 2013). Notably, feeling unprepared for the emotional consequences of ethically challenging decision making and exposure to incidents where the rules of engagement are unclear may potentially be unique risk factors for experiencing military-related moral injury. In the present study, tailored changes to pre-operational briefings were thought to have the potential to protect personnel from moral injury-related distress. Previous research has found that pre-deployment briefings can have protective effects against later psychological distress during deployment (Mulligan et al., 2010). It is possible that additional pre-deployment preparation about the ethically challenging decisions personnel may face and clarifications of the rules of engagement, as well as a tailored debrief following a PMIE, may safeguard against moral injury-related distress. Research in civilian medic samples suggests that supportive discussions with senior colleagues who share their own workplace difficulties can help juniors to reflect on their own challenges and mitigate feelings of shame (Miles, 2019). However, further research is needed to explore the role personnel briefings and guidance from the chain of command may play in moral injury.

### Limitations

A strength of this study is the diverse nature of the clinician sample, which included those with both military and civilian backgrounds recruited from a range of UK mental health services. Clinicians had also provided treatment to Armed Forces personnel or Veteran patients following a variety of morally injurious events. As study inclusion criteria required clinicians to have provided treatment to service personnel or a Veteran within the last six months, a possible limitation is the exclusion of clinicians with less experience in the identification and treatment of moral injury-related mental health problems. It was also beyond the scope of this study to examine whether clinicians perceived there to be differences in treating active service personnel compared to ex-service personnel affected by moral injury and this should be examined in future studies. Given the qualitative nature of the study, a large-scale UK quantitative investigation would be useful in determining the generalizability of the findings and how they compare across clinical settings internationally. Finally, as exposure to PMIEs is not unique to service personnel and Veterans, it may be beneficial in future studies to include of the views of clinicians who provide psychological intervention to other similarly exposed groups, such as first responders or journalists (Williamson et al., 2018) to further our theoretical understanding of moral injury and its treatment.

## Conclusions

This research expands on earlier qualitative studies (Drescher et al., 2011; Williamson et al., 2019) and provides further insight into the experiences of UK clinicians providing care to military personnel and Veterans who may be affected by moral injury-related psychological problems, detailing the specific treatment approaches currently utilized. Further, these findings highlight the range of difficulties faced by clinicians when working with patients affected by moral injury, including dealing with challenging symptomology and the impact of providing such treatment on the therapist’s own mental health. Future research is needed to evaluate the effectiveness of the treatments currently provided for mental health problems following moral injury. This, as well as the development of clear guidance on best practice for treating moral injury-related mental health difficulties in UK personnel/Veterans, may improve clinician confidence in delivering care to those adversely impacted by PMIEs. Finally, this study indicates the possible risk and protective factors for experiencing moral injury related distress and further studies are needed to examine pathways for prevention and intervention for personnel/Veterans who have experienced a morally injurious event.

## Supplementary Material

Supplemental Material

## Author statement

VW, DM, SAMS, SA, EJ, NG made substantial contributions to the conception of the study and interpretation of data for the study; drafted the article and revised it critically for important intellectual content; gave final approval of the version to be published and agreed to be accountable for all aspects of the article in ensuring that questions related to the accuracy or integrity of any part of the article are appropriately investigated and resolved. VW and SA contributed to acquisition analysis of the data.

## Data sharing statement

No additional data are available.
