# Supplementary material for: Delivering treatment to morally injured UK military personnel and Veterans: The clinician experience
Source: Mil Psychol. 2021 Mar 29;33(2):115–23. doi: 10.1080/08995605.2021.1897495 (PMC10013382; doi:10.1080/08995605.2021.1897495)
Supplement: Supplemental Material [file HMLP_A_1897495_SM4460.docx]

Supplementary File 1

*Topic guide for semi-structured interviews with clinicians*

| What do you know about the concept of ‘moral injury’? |
| --- |
| How would you define it? |
| How do you think MI might be different to other traumas involving threats to self or others? |
| Do you think the term ‘moral injury’ is a good descriptor or would you prefer a different term/expression? |
| Have you provided ongoing care with service personnel or veterans who were exposed to morally injurious events? |
| What types of experiences do morally injured veterans present with? |
| Is there a particular type of morally injurious experience that is especially distressing for personnel/veterans? |
| When personnel/veterans recollect the morally injurious event, what cognitions do they report? |
| What emotions do they report? |
| What physical symptoms do they report? |
| How does this compare to a personnel/veteran exposed to other types of trauma? |
| Has their morally injurious experience changed how they view themselves as a person?  Why or why not? |
| How do you think this is the same/different to those exposed to other kinds of trauma? |
| Has the event impacted how personnel/veterans make sense of life and its meaning? |
| Has it affected their spirituality or religious beliefs? |
| Has the event changed how they think about the future? |
| How do they see the future now? |
| Has the event had any impact on their ability to make plans for the future? |
| Has the event affected their relationships with others? |
| Has the event effected how they care for other people? |
| Has the event impacted their trust in other people? |
| Has the event had any impact on their work? |
| Are personnel/veterans who have these experiences typically employed? |
| Has the experience impacted how they perform in their job? |
| How has the event impacted their relationships with their boss/colleagues? |
| How do their moral injury related difficulties affect their daily life? |
| Has their experience changed how they care for themselves? |
| What is their physical health like? |
| Are there any factors that might make some personnel/veterans more/less likely to feel distressed on exposure to morally injurious events? |
| Are there any pre-event risk factors? During event itself? After the event? |
| Are there any protective factors before/during/after the event that might make someone less likely to be affected? |
| What might lead you to consider that a patient might have experienced a moral injury? |
| How have you approached working with service members/veterans to address their moral injury-related issues? |
| How does this approach compare to treatment for individuals with other trauma types? |
| How do you feel about using this approach? |
| What are some of the challenges of working with service members/veterans to address symptoms following experiences of moral injury? |
| Typically, how many treatment sessions do they need? How does this compare to non-morally injured populations? |
| How do you manage the ethical implications of disclosures of morally injurious experiences by veterans? |
| Is anything needed to better promote recovery among service members/veterans following moral injury? |
| At a service level? |
| At a policy or government level? |
| In terms of clinician training? |
| What is it like for you personally to provide treatment in cases for moral injury? |
| Is it the same/different to working with individuals exposed to other trauma types? |
| What supervision or support do you currently receive in relation to your clinical work? |
| Is there any other support or training you would find helpful? |
